# MR guided focused ultrasound as a treatment tool for essential and Parkinsonian tremor

**DOI:** 10.1186/2050-5736-3-S1-O3

**Published:** 2015-06-30

**Authors:** Menashe Zaaroor, Alon Sinai, Ayelet Eran, Dorit Goldsher, Ilana Erikh, Maria Nassar, Ilana Schlesinger

**Affiliations:** 1 Rambam Health Care Campus, Haifa, Israel

## Background/introduction

Combining focused ultrasound (FUS) with real time MRI guidance and monitoring enables a new non-invasive treatment of brain disorders. In this technique multisource ultrasound waves are focused at a single point raising the temperature gradually until ablation occurs. Lesions as small as 2-4 mm can be made, without impact on surrounding tissue or remote brain tissue.

Our aim was to assess MR guided focused ultrasound (MRgFUS) as a new non-invasive surgical tool for treating essential and parkinsonian tremor by thalamotomy of the ventral-intermediate nucleus.

## Methods

Eight patients with severe medically refractory tremor, 4 essential tremor and 4 with Parkinsonian tremor, underwent unilateral ventral-intermediate nucleus thalamotomy using MRgFUS. Following the preoperative planning using 3D CT and 3D 3T MRI, patients lay in the MRI scanner while awake with their head immobilized in a stereotactic frame. The transducers helmet was mechanically positioned and a silicone membrane sealed the space between the patient’s head and the helmet.

MRI scans was performed with the helmet and frame in place, the target identified and accuracy verified at low temperatures. Treatment consisted of multiple sonications at gradually increased temperatures. MRI thermal scan provided feedback on the temperature achieved and the target accuracy. Between sonications patients provided information concerning side effects and neurological examinations were performed to verify effect.

## Results

Tremor stopped in the contralateral upper extremity in all patients immediately following the procedure. In two Parkinson disease patients ipsilateral rigidity was decreased as well. Immediate side effects included during sonications: vomiting (n=1), and transient forehead pain (2), transient vertigo (2) and following the procedure: scalp numbness (transient n=2, for 1day-1 week, permanent- mild n=1), subjective transient gait unsteadiness (n=3, for 1 week) Clinical assessment of the examiner and patient changed from severe disability to no functional disability from tremor immediately following the procedure. Follow-up of up to 10 months show sustained effect. No late side effects were noted.

## Conclusions

Thalamotomy of the VIM using MRIgFUS is safe and effective in both Parkinson disease tremor and essential tremor with minimal mostly transient side effects. Large randomized studies and longer follow up are needed in order to assess safety and efficacy.

